# Relationship Between *LTA4H* Promotor Polymorphism and Tuberculosis-Associated Immune Reconstitution Inflammatory Syndrome and Its Prevention With Prednisone

**DOI:** 10.1093/ofid/ofad379

**Published:** 2023-07-17

**Authors:** Cari Stek, Muki Shey, Khuthala Mnika, Charlotte Schutz, Friedrich Thienemann, Robert J Wilkinson, Lutgarde Lynen, Graeme Meintjes

**Affiliations:** Wellcome Center for Infectious Diseases Research in Africa, Institute of Infectious Disease and Molecular Medicine, University of Cape Town, Cape Town, South Africa; Department of Clinical Sciences, Institute of Tropical Medicine, Antwerp, Belgium; Department of Medicine, University of Cape Town, Cape Town, South Africa; Wellcome Center for Infectious Diseases Research in Africa, Institute of Infectious Disease and Molecular Medicine, University of Cape Town, Cape Town, South Africa; Department of Medicine, University of Cape Town, Cape Town, South Africa; Division of Human Genetics, Department of Pathology, University of Cape Town, Cape Town, South Africa; Wellcome Center for Infectious Diseases Research in Africa, Institute of Infectious Disease and Molecular Medicine, University of Cape Town, Cape Town, South Africa; Department of Medicine, University of Cape Town, Cape Town, South Africa; Wellcome Center for Infectious Diseases Research in Africa, Institute of Infectious Disease and Molecular Medicine, University of Cape Town, Cape Town, South Africa; Department of Medicine, University of Cape Town, Cape Town, South Africa; Wellcome Center for Infectious Diseases Research in Africa, Institute of Infectious Disease and Molecular Medicine, University of Cape Town, Cape Town, South Africa; Department of Medicine, University of Cape Town, Cape Town, South Africa; Francis Crick Institute, London, United Kingdom; Department of Infectious Diseases, Imperial College London, London, United Kingdom; Department of Clinical Sciences, Institute of Tropical Medicine, Antwerp, Belgium; Wellcome Center for Infectious Diseases Research in Africa, Institute of Infectious Disease and Molecular Medicine, University of Cape Town, Cape Town, South Africa; Department of Medicine, University of Cape Town, Cape Town, South Africa

**Keywords:** corticosteroids, immune reconstitution inflammatory syndrome, IRIS, *LTA4H* genotype, tuberculosis

## Abstract

The development of paradoxical tuberculosis-associated immune reconstitution inflammatory syndrome (TB-IRIS) and its prevention using prednisone may potentially be mediated by the *LTA4H* genotype. We assessed this hypothesis in a clinical trial evaluating prednisone to prevent TB-IRIS. We did not find an association between *LTA4H* genotype and TB-IRIS incidence or prednisone efficacy.

Paradoxical tuberculosis-associated immune reconstitution inflammatory syndrome (TB-IRIS), an immunopathological reaction resulting in new, recurrent, or worsening signs or symptoms of tuberculosis (TB), complicates the initiation of antiretroviral therapy (ART) in approximately 18% (95% confidence interval [CI], 16%–21%) of patients being treated for TB [1]. Prednisone has been shown to be efficacious for both treatment and prevention of TB-IRIS [2, 3]; this efficacy may be associated with the *LTA4H* genotype [4]. LTA4 hydrolase (LTA4H) is an enzyme that hydrolyzes leukotriene A4 into the proinflammatory LTB4. Its expression is regulated by a single-nucleotide polymorphism (SNP) close to the promoter region of the *LTA4H* gene [5]. The CC genotype is associated with lower concentrations of LTA4H, whereas the TT genotype is associated with higher LTA4H concentrations and activity [5]. Higher production of proinflammatory cytokines in CT/TT genotypes could play a role in the development of TB-IRIS. One study evaluated *LTA4H* genotype in TB-IRIS in an Indian cohort of 142 patients with newly diagnosed TB and low CD4 cell counts; it found an association between genotype and incidence of TB-IRIS, though only for severe TB-IRIS (defined as a Karnofsky score of ≤50 or a clinical condition mandating hospitalization or prolonging of hospital admission) [6]. Corticosteroids were similarly effective in treating TB-IRIS across all genotypes [6], contrasting with prior findings in tuberculous meningitis (TBM) where treatment with corticosteroids was only beneficial in those with the hyperinflammatory TT genotype [4].

In this study, we assessed the association of *LTA4H* genotype with the development of TB-IRIS, several plasma chemokines and cytokines, and the efficacy of prednisone for preventing TB-IRIS.

## METHODS

This was a substudy of the PredART trial [3]. In this randomized, double-blind, placebo-controlled trial, adult patients identified as being at high risk for paradoxical TB-IRIS (time between initiation of anti-TB treatment and ART <30 days and CD4 count **≤**100 cells/μL) were randomized to receive prednisone (40 mg daily for 2 weeks followed by 20 mg daily) or identical placebo during the first 4 weeks of ART. TB-IRIS was the primary endpoint and was adjudicated by a committee of 3 independent clinical experts using the International Network for the Study of HIV-Associated IRIS case definition [7]. Prophylactic prednisone reduced the incidence of TB-IRIS by 30%, without an excess of adverse events [3].

DNA was extracted from whole blood using QIAamp DNA Blood Midi Kit (Qiagen, Hilden, Germany) and stored at −20°C. The *LTA4H* gene promoter region SNP (rs17525495) was genotyped using a TaqMan SNP Genotyping Assay and TaqMan Universal Master Mix (Life Technologies, Carlsbad, California); validation was done in a subset of the sample (10%) by Sanger sequencing. Stored plasma samples collected at week 0 and week 2 were used to measure several chemokines and cytokines, using Bio-Plex Pro Human Cytokine 27-plex Assay (Bio-Rad, Hercules, California) and standardized enzyme-linked immunosorbent assays (R&D Systems, Minneapolis, Minnesota) according to the manufacturer's instructions.

*LTA4H* genotype is presented as CC and CT/TT. Consistency of the observed genotypes with the Hardy-Weinberg equilibrium and association between *LTA4H* genotype and development of TB-IRIS were assessed using the Pearson χ^2^ test. Concentrations of chemokines and cytokines were compared between CC and CT/TT genotypes using the Wilcoxon rank-sum test. The *P* value signifying significance was adjusted for multiple comparisons using the Bonferroni correction. The effect of *LTA4H* genotype on the efficacy of prophylactic prednisone was assessed using Cox proportional hazard models and represented in Kaplan-Meier plots, with time to TB-IRIS as outcome and treatment arm, genotype, and their interaction as variables.

### Ethical Considerations

The PredART trial obtained ethics approval from the University of Cape Town Human Research Ethics Committee (HREC 136/2013), the Institute of Tropical Medicine Institutional Review Board (882/13), and the Antwerp University Hospital Ethical Committee (13/20/224). Written informed consent and separate genetic informed consent were obtained for all participants of this substudy.

## RESULTS

*LTA4H* genotyping was available for 213 of 240 trial participants; reasons for missing data are listed in Supplementary Table 1. Baseline characteristics were similar to baseline characteristics of the whole PredART trial study population: 60% were male, median age was 37 years (interquartile range [IQR], 30–43 years), and median CD4 count was 48 cells/μL (IQR, 23–85 cells/μL). Eighty-three participants (39%) developed TB-IRIS at a median of 9 days (IQR, 5–13 days) after starting ART.

Among the 213 participants, 173 (81%) had a CC, 31 (15%) had a CT, and 9 (4%) had a TT genotype. The overall observed distribution of alleles roughly resembles the African distribution [8]; however, genotypes were not in Hardy-Weinberg equilibrium (*P* < .0001). Because of the low frequency of CT and TT genotypes, we grouped these for further analysis. We found no association between genotype and the development of TB-IRIS: TB-IRIS occurred in 67 participants (39%) in the CC group and 16 participants (40%) in the CT/TT group. Comparing the 3 genotypes separately showed similar results (Supplementary Table 2). We also did not find an association between genotype and the development of TB-IRIS when only assessing participants in the placebo arm (44% vs 52%; *P* = .51), nor did we find an association between genotype and more severe TB-IRIS (defined as TB-IRIS symptoms sufficient to lead the clinical investigator to prescribe open-label corticosteroid treatment) (20% vs 25%; *P* = .51). Next, we compared different chemokines and cytokines between participants with CC and CT/TT *LTA4H* genotype. We found no association between genotype and concentrations of these analytes at week 0 or week 2 in the entire cohort (Supplementary Table 3), nor at week 2 in participants in the placebo arm only (Supplementary Table 4).

**Figure 1.**
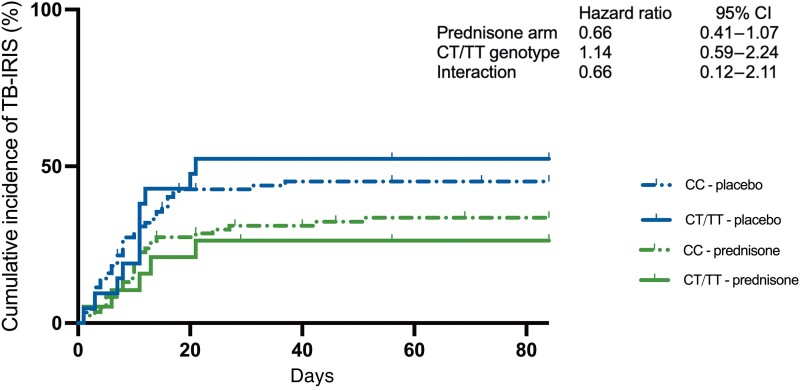
Cumulative incidence of tuberculosis-associated immune reconstitution inflammatory syndrome (TB-IRIS) by *LTA4H* genotype and study arm. The effect of *LTA4H* genotype on the efficacy of prophylactic prednisone was assessed using Cox proportional hazard models with time to TB-IRIS as outcome and treatment arm, genotype, and their interaction as variables. Abbreviations: CI, confidence interval; TB-IRIS, tuberculosis-associated immune reconstitution inflammatory syndrome.

Last, we assessed whether *LTA4H* genotype affects the efficacy of prednisone to prevent TB-IRIS. Although prednisone appeared to prevent TB-IRIS more effectively in those with a CT/TT genotype (hazard ratio, 0.66 [95% CI, .21–2.11]), the difference was not statistically significant (*P* = .49) and the wide confidence interval prevented us from making any conclusions regarding efficacy in relation to genotype (Figure 1). Repeating the analyses comparing those with a TT genotype with those with CC and CT genotype combined showed similar results (Supplementary Table 5).

## DISCUSSION

We could not show an association between *LTA4H* genotype and TB-IRIS incidence or prednisone efficacy in our study population. The allelic distribution was not in Hardy-Weinberg equilibrium. An explanation for the lower-than-expected proportion of heterozygotes in our cohort could be the large proportion of participants with extrapulmonary TB; in some studies the CT genotype was associated with a decreased risk for extrapulmonary TB [5, 9], although this association has not been found in other studies [9–11].

LTA4H activity influences LTB4 activity. LTB4 attracts neutrophils and macrophages to sites of inflammation; in cerebrospinal fluid (CSF), *LTA4H* genotype is associated with concentrations of inflammatory cytokines [12]. Therefore, we hypothesized that *LTA4H* genotype would be associated with inflammation seen in TB-IRIS. However, similar to 2 other studies assessing LTA4H and TB-IRIS [6] or TB paradoxical reactions [13], we did not find an association between *LTA4H* genotype and TB-IRIS, nor did we find an association between *LTA4H* genotype and chemokine and cytokine profiles. There are several possible reasons why we did not detect this association. First, there may be a difference in the role of LTA4H in human immunodeficiency virus (HIV)–positive and HIV-negative individuals: in the above-mentioned study [12], *LTA4H* genotype did not affect CSF cytokine concentrations in HIV-positive patients, and a survival benefit for those with a TT genotype was also only evident for HIV-negative patients. Moreover, HIV affects the ability of neutrophils [14] and alveolar macrophages [15] to produce LTB4 in vitro, and LTB4—usually elevated in bronchoalveolar lavage fluid of patients with bacterial pneumonia—was not elevated in HIV-positive patients with pneumonia compared to healthy controls [16]. Second, there are important differences in pathogenesis between pulmonary TB and TBM: studies showing an association between clinical presentation or outcome and *LTA4H* genotype all relate to TBM [5, 9], whereas studies assessing pulmonary TB did not find this association [9, 11]. LTA4H is not only involved in generation of the proinflammatory LTB4, it also breaks down proline-glycine-proline (PGP) [17], a tripeptide that is generated from collagen by matrix metalloproteinases upregulated in TB. PGP attracts neutrophils and plays a role in inflammatory lung disease [18]. Although our trial included many participants with extrapulmonary TB, those with TBM were excluded. If the anti-inflammatory role of LTA4H—through breaking down PGP—is more prominent in pulmonary TB compared with its role in TBM, this might explain our findings.

We did not find a statistically significant effect of *LTA4H* genotype on the efficacy of prednisone to prevent TB-IRIS. This could be due to the low frequency of the T allele in our African study population (10%) compared to studies performed in Southeast Asia, where this allele is more frequent (33%) (https://www.ensembl.org/index.html). However, a study done in South India, in which 40% of the participants bore the T allele, also did not find the response to steroids (used as treatment for TB-IRIS, not as prophylaxis) to be genotype dependent [6].

In conclusion, in our study, *LTA4H* genotype was not associated with the development of TB-IRIS. Moreover, we were unable to show that the efficacy of prednisone to prevent TB-IRIS is genotype dependent.

## Supplementary Material

ofad379_Supplementary_Data

## Supplementary Data

Supplementary materials are available at *Open Forum Infectious Diseases* online. Consisting of data provided by the authors to benefit the reader, the posted materials are not copyedited and are the sole responsibility of the authors, so questions or comments should be addressed to the corresponding author.

## Notes

***Open access statement.*** For the purpose of open access, the author applied a CCBY public copyright licence to any author-accepted manuscript arising from this submission.

***Financial support.*** This work was supported by the European and Developing Countries Clinical Trials Partnership through a Strategic Primer Grant (SP.2011.41304.074) awarded to the University of Cape Town, the Institute of Tropical Medicine, and Imperial College London. This work also received funding from the South African Government Department of Science and Technology (grant number 64787); the Wellcome Trust (grant numbers 098316 and 203135); and a PhD fellowship from the Institute of Tropical Medicine. R. J. W. is supported by the Francis Crick Institute.
