# Supplementary material for: Relationship Between LTA4H Promotor Polymorphism and Tuberculosis-Associated Immune Reconstitution Inflammatory Syndrome and Its Prevention With Prednisone
Source: Open Forum Infect Dis. 2023 Jul 17;10(7):ofad379. doi: 10.1093/ofid/ofad379 (PMC10375423; doi:10.1093/ofid/ofad379)
Supplement: ofad379_Supplementary_Data [file ofad379_supplementary_data.docx]

**Supplementary tables**

| TABLE S1 Reasons for missing participant data | |
| --- | --- |
| Never received a dose of prednisone | 2 |
| No genetics consent obtained | 1 |
| DNA extraction unsuccessful | 18 |
| Logistical reasons | 6 |

| TABLE S2 Participants developing TB-IRIS per genotype | | | |
| --- | --- | --- | --- |
| genotype  TB-IRIS | CC | CT | TT |
| Yes (n; %) | 106 (61) | 17 (55) | 7 (78) |
| No (n; %) | 67 (39) | 14 (45) | 2 (22) |
|  |  |  | **P = 0.46** |
|  | | | |
| Numbers were compared using the Pearson chi-square test; a p-value of < 0.05 was considered statistically significant | | | |

| TABLE S3 Cytokine concentrations comparing CC vs CT/TT *LTA4H* genotype at week 0 (start ART) and week 2. | | | | | | | | | | |
| --- | --- | --- | --- | --- | --- | --- | --- | --- | --- | --- |
|  |  | | | |  |  | | | | |
|  | **Week 0** | | | |  | **Week 2** | | | |  |
|  | **CC genotype (n = 124)** | | **CT/TT genotype (n = 32)** | | **p-value** | **CC genotype (n = 105)** | | **CT/TT genotype (n = 28)** | | **p-value** |
|  |  |  |  |  |  |  |  |  |  |  |
| **IL-1b** | 0.12 | (0.11-0.14) | 0.14 | (0.12-0.18) | 0.03 | 0.12 | (0.11-0.16) | 0.15 | (0.11-0.2) | 0.16 |
| **IL-18** | 1125.8 | (793.2-1946.9) | 901.0 | (647.9-1629.1) | 0.14 | 890.1 | (564.7-1333.2) | 894.6 | (506.5-1493.3) | 0.72 |
| **IL-1ra** | 112.7 | (73.7-235.4) | 116.8 | (91.2-267.3) | 0.60 | 122.8 | (73.5-299.8) | 157.5 | (90.0-520.6) | 0.28 |
| **IL-6** | 0.10 | (0.09-0.31) | 0.10 | (0.07-0.24) | 0.66 | 0.14 | (0.09-0.61) | 0.12 | (0.09-0.42) | 0.85 |
| **TNF** | 14.1 | (9.7-22.9) | 18.5 | (14.0-25.1) | 0.03 | 17.9 | (9.1-32.4) | 20.2 | (9.8-30.6) | 0.39 |
| **IFN-y** | 0.36 | (0.32-0.86) | 0.39 | (0.34-0.90) | 0.73 | 0.36 | (0.19-0.55) | 0.36 | (0.24-0.65) | 0.33 |
| **IL-12p70** | 0.33 | (0.25-0.41) | 0.34 | (0.25-0.36) | 0.91 | 0.34 | (0.25-0.40) | 0.33 | (0.25-0.36) | 0.44 |
| **IL-17** | 0.46 | (0.34-0.51) | 0.34 | (0.25-0.55) | 0.36 | 0.46 | (0.34-0.58) | 0.46 | (0.28-0.79) | 0.99 |
| **IL-2** | 1.07 | (0.62-1.59) | 1.28 | (0.79-2.27) | 0.17 | 1.22 | (0.62-2.11) | 1.06 | (0.63-2.39) | 0.95 |
| **IL-7** | 1.40 | (1.14-1.64) | 1.47 | (1.14-2.48) | 0.33 | 1.40 | (1.14-1.80) | 1.47 | (1.40-2.39) | 0.10 |
| **IL-4** | 0.15 | (0.08-0.54) | 0.18 | (0.08-0.46) | 0.95 | 0.14 | (0.08-0.51) | 0.22 | (0.14-0.46) | 0.24 |
| **IL-9** | 24.4 | (19.5-32.5) | 28.3 | (22.1-31.4) | 0.26 | 23.7 | (19.4-30.4) | 26.1 | (23.2-30.3) | 0.14 |
| **IL-13** | 0.05 | (0.05-0.07) | 0.05 | (0.05-0.06) | 0.32 | 0.05 | (0.05-0.07) | 0.05 | (0.05-0.06) | 0.40 |
| **IL-10** | 0.41 | (0.31-0.58) | 0.41 | (0.31-0.54) | 0.84 | 0.41 | (0.31-0.58) | 0.41 | (0.31-0.95) | 0.48 |
| **CCL2** | 12.4 | (5.8-25.8) | 16.5 | (6.9-26.1) | 0.46 | 8.14 | (2.70-17.81) | 9.82 | (5.42-20.25) | 0.15 |
| **CCL3** | 1.92 | (1.15-3.35) | 2.00 | (1.12-3.89) | 0.88 | 2.25 | (1.39-5.63) | 2.15 | (1.10-6.36) | 0.67 |
| **CCL4** | 24.4 | (19.4-30.0) | 25.5 | (21.6-31.2) | 0.27 | 26.1 | (21.5-33.9) | 28.8 | (23.9-39.4) | 0.18 |
| **CCL5** | 1289.6 | (572.5-2764.4) | 1178.8 | (477.7-2692.1) | 0.68 | 1208.2 | (546.7-1953.0) | 1082.6 | (496.5-2414.1) | 0.94 |
| **CCL11** | 9.43 | (4.45-18.14) | 8.62 | (5.29-16.35) | 0.94 | 8.35 | (4.38-20.8) | 11.2 | (6.0-17.4) | 0.55 |
| **CXCL8** | 1.21 | (0.91-3.64) | 1.32 | (1.06-4.25) | 0.50 | 1.40 | (0.91-4.61) | 1.09 | (1.02-3.98) | 0.84 |
| **CXCL10** | 997.2 | (406.0-1595.3) | 848.1 | (552.9-1636.4) | 0.86 | 602.5 | (281.7-1326.9) | 675.4 | (361.6-1406.0) | 0.59 |
| **PDGF** | 20.1 | (11.9-50.4) | 21.3 | (11.5-37.4) | 0.63 | 22.6 | (13.4-52.7) | 30.4 | (15.7-57.8) | 0.31 |
| **basic FGF** | 1.95 | (1.29-3.31) | 2.04 | (1.29-2.85) | 0.79 | 1.94 | (1.29-2.93) | 2.02 | (1.29-2.70) | 0.71 |
| **CD14** | 4285.8 | (3347.3-5401.3) | 4404.7 | (3397.3-5772.0) | 0.61 | 4162.0 | (3028.7-5294.1) | 4195.2 | (3314.1-5531.0) | 0.82 |
| **CD163** | 1063.3 | (822.0-1462.9) | 1015.9 | (711.7-1474.9) | 0.54 | 1039.6 | (727.6-1491.8) | 986.1 | (727.6-1296.6) | 0.77 |
| **TF** | 37.3 | (30.8-43.6) | 34.9 | (29.5-46.2) | 0.56 | 38.7 | (30.7-45.9) | 37.6 | (31.1-49.2) | 0.69 |
|  |  |  |  |  |  |  |  |  |  |  |
| Data are shown as median (interquartile range). Concentrations (pg/ml) were compared using the Wilcoxon rank sum test. A p-vaule < 0.002 was considered significant having adjusted for multiple comparisons using the Bonferroni correction. | | | | | | | | | | |

| TABLE S4 Cytokine concentrations comparing CC vs CT/TT *LTA4H* genotype at week 2 in the placebo arm | | | | | | | | | | |
| --- | --- | --- | --- | --- | --- | --- | --- | --- | --- | --- |
|  |  | | | |  |  | | | | |
|  | **Week 0** | | | |  | **Week 2** | | | |  |
|  | **CC genotype (n = 63)** | | **CT/TT genotype (n = 16)** | |  | **CC genotype (n = 54)** | | **CT/TT genotype (n = 14)** | | **p-value** |
|  |  |  |  |  |  |  |  |  |  |  |
| **IL-1b** | 0.12 | 0.11 - 0.14 | 0.14 | 0.13 - 0.19 |  | 0.13 | 0.11 - 0.18 | 0.12 | 0.09 - 0.16 | 0.09 |
| **IL-18** | 1188.85 | 877.97 - 2085.33 | 949.54 | 695.49 - 1748.20 |  | 972.59 | 598.47 - 2163.10 | 967.07 | 473.01 - 1445.70 | 0.89 |
| **IL-1ra** | 122.10 | 67.44 - 234.08 | 162.52 | 86.33 - 267.31 |  | 187.40 | 84.26 - 468.10 | 123.30 | 63.19 - 198.14 | 0.31 |
| **IL-6** | 0.10 | 0.09 - 0.66 | 0.10 | 0.07 - 0.22 |  | 0.28 | 0.09 - 1.12 | 0.10 | 0.09 - 0.18 | 0.96 |
| **TNF** | 14.65 | 10.10 - 24.44 | 22.35 | 14.04 - 27.70 |  | 20.82 | 10.61 - 37.60 | 15.14 | 8.82 - 30.22 | 0.41 |
| **IFN-y** | 0.37 | 0.33 - 0.77 | 0.42 | 0.34 - 0.93 |  | 0.36 | 0.19 - 0.60 | 0.35 | 0.11 - 0.65 | 0.27 |
| **IL-12p70** | 0.34 | 0.25 - 0.40 | 0.36 | 0.29 - 0.36 |  | 0.36 | 0.25 - 0.40 | 0.25 | 0.25 - 0.36 | 0.94 |
| **IL-17** | 0.46 | 0.34 - 0.61 | 0.40 | 0.25 - 0.67 |  | 0.46 | 0.34 - 0.62 | 0.40 | 0.34 - 0.49 | 0.89 |
| **IL-2** | 1.03 | 0.62 - 1.54 | 1.83 | 1.19 - 2.51 |  | 1.26 | 0.62 - 2.25 | 1.06 | 0.62 - 1.99 | 0.79 |
| **IL-7** | 1.40 | 1.14 - 1.80 | 1.43 | 1.09 - 3.00 |  | 1.40 | 1.37 - 1.70 | 1.53 | 1.40 - 2.39 | 0.46 |
| **IL-4** | 0.14 | 0.08 - 0.55 | 0.32 | 0.08 - 0.50 |  | 0.14 | 0.09 - 0.50 | 0.15 | 0.14 - 0.30 | 0.42 |
| **IL-9** | 23.83 | 18.43 - 32.82 | 27.06 | 23.78 - 31.38 |  | 25.40 | 20.77 - 30.56 | 24.64 | 21.57 - 30.85 | 0.35 |
| **IL-13** | 0.05 | 0.05 - 0.07 | 0.05 | 0.05 - 0.06 |  | 0.05 | 0.05 - 0.07 | 0.05 | 0.05 - 0.06 | 0.16 |
| **IL-10** | 0.41 | 0.31 - 0.48 | 0.41 | 0.31 - 1.27 |  | 0.41 | 0.31 - 0.63 | 0.41 | 0.41 - 1.09 | 0.74 |
| **CCL2** | 13.17 | 5.91 - 27.79 | 21.18 | 13.30 - 32.50 |  | 7.08 | 3.38 - 17.94 | 5.80 | 3.59 - 18.73 | 0.03 |
| **CCL3** | 1.78 | 1.08 - 3.19 | 2.21 | 1.57 - 5.91 |  | 2.67 | 1.78 - 6.19 | 2.04 | 1.11 - 4.26 | 0.63 |
| **CCL4** | 24.37 | 19.42 - 29.30 | 25.80 | 22.57 - 28.23 |  | 28.17 | 23.20 - 36.78 | 28.39 | 20.92 - 43.07 | 0.94 |
| **CCL5** | 1256.41 | 492.83 - 3012.26 | 870.29 | 433.34 - 1489.86 |  | 1212.62 | 546.66 - 2134.79 | 1082.62 | 453.73 - 1593.38 | 0.79 |
| **CCL11** | 7.65 | 3.68 - 17.29 | 12.94 | 6.51 - 18.15 |  | 6.62 | 3.72 - 17.80 | 6.94 | 3.77 - 15.55 | 0.11 |
| **CXCL8** | 1.15 | 0.61 - 3.24 | 2.15 | 1.06 - 8.03 |  | 1.55 | 1.02 - 5.75 | 1.06 | 0.64 - 1.32 | 0.76 |
| **CXCL10** | 893.81 | 385.18 - 1594.31 | 895.26 | 605.83 - 2006.16 |  | 786.55 | 333.44 - 1425.52 | 414.86 | 271.52 - 764.42 | 0.20 |
| **PDGF** | 17.62 | 11.64 - 46.38 | 24.05 | 12.92 - 37.42 |  | 23.94 | 14.84 - 54.36 | 23.25 | 11.64 - 45.68 | 0.12 |
| **basic FGF** | 1.94 | 1.29 - 2.29 | 2.38 | 1.38 - 3.16 |  | 1.82 | 1.29 - 2.77 | 1.55 | 1.29 - 2.62 | 0.48 |
| **CD14** | 4348.29 | 3427.46 - 5320.89 | 5704.61 | 4428.97 - 6423.65 |  | 4594.51 | 3530.97 - 5530.36 | 3828.74 | 2929.33 - 5409.18 | 0.86 |
| **CD163** | 1087.21 | 888.32 - 1481.93 | 881.36 | 521.62 - 1449.35 |  | 1139.88 | 751.92 - 1780.13 | 1037.82 | 727.56 - 2096.02 | 0.13 |
| **TF** | 37.08 | 31.21 - 43.59 | 39.87 | 32.13 - 51.71 |  | 37.16 | 30.60 - 43.90 | 37.16 | 31.21 - 52.05 | 0.34 |
|  |  |  |  |  |  |  |  |  |  |  |
| Data are shown as median (interquartile range). Concentrations (pg/ml) were compared using the Wilcoxon rank sum test. A p-vaule < 0.002 was considered significant having adjusted for multiple comparisons using the Bonferroni correction. Week 0 values are only given for reference. | | | | | | | | | | |

| TABLE S5 Efficacy of prednisone to prevent TB-IRIS comparing TT vs CT/CC genotype | | |
| --- | --- | --- |
|  | Hazard ratio | 95% confidence interval |
| Prednisone arm | 0.64 | 0.41 – 1.00 |
| TT genotype | 1.21 | 0.16 – 8.77 |
| Interaction term | 0.34 | 0.21 – 5.67 |
|  |  |  |
| Assessment was done using Cox proportional hazard models | | |
